# Emotional activation in a cognitive behavioral setting: extending the tradition with embodiment

**DOI:** 10.3389/fpsyg.2024.1409373

**Published:** 2024-07-25

**Authors:** Gernot Hauke, Christina Lohr-Berger, Tal Shafir

**Affiliations:** ^1^Embodiment Resources Academy, Munich, Germany; ^2^The Graduate School of Creative Arts Therapies, Faculty of Social Welfare and Health Sciences, University of Haifa, Haifa, Israel

**Keywords:** embodiment, emotional activation, emotion regulation, CBT, bodily expressions of emotions, levels of emotional awareness, interoception

## Abstract

The neuroscience-based concept of “embodied cognition” or “embodiment” highlights that body and psyche are closely intertwined, i.e., effects of body and psyche are bidirectional and reciprocal. This represents the view that cognitive processes are not possible without the direct participation of the body. Traditional Cognitive Behavioral Therapy (CBT) addresses emotional processes on a conceptual level (dysfunctional thoughts, beliefs, attributions, etc.). However recent findings suggest that these processes already start at the level of bodily sensations. This opens up a way of working in therapy that includes the level of bodily sensations, where the development of emotional meaning is supported by bottom-up processes. Bidirectionality of embodiment can be effectively exploited by using body postures and movements associated with certain emotions, which we refer to as embodiment techniques, to deepen the physical experience of poorly felt emotions and support the valid construction of emotional meaning. This embodied approach offers several advantages: Prelinguistic or hard-to-grasp aspects can be identified more easily before being processed verbally. It is also easier to work with clients who have limited access to their emotions. Thus, in this paper we describe a new embodied CBT approach to working on the dysfunctional schema, which is based on three modules: body focus, emotional field, and interaction focus. In addition, using specific zones in the space of the therapy-room allows the embodiment of problematic interactions, as well as of power and powerlessness, closeness and distance, etc. Directly experiencing these processes on one’s own body in the protected space of therapy allows faster and deeper insights than would be possible with conversations alone. Finally, the vitalizing power of emotions is used to create coherent action plans and successful interactions. This working method is illustrated by means of a case from practice.

## 1 Introduction

In a review article “Embodiment in Clinical Disorders and Treatment” Riskind et al. recognize Cognitive Behavioral Therapy (CBT) as a successful treatment method, which is considered the preferred “gold standard” treatment for many mental disorders (Riskind et al., 2021). However, these authors also cite findings showing that a significant percentage of patients do not respond to this treatment or relapse later. They suggest that among several factors that might explain this phenomenon, insufficient attention to the embodiment of mental states may play an important role.

Classical CBT is concerned with the cognitive concepts of self and world-view and the resulting expectations, beliefs, attributions, etc. (Beck, 2008; Beck and Haigh, 2014). Consistent with this approach, it refers also to emotional processes at a conceptual level (Beck and Haigh, 2014). However, in more recent approaches to understanding cognition, it is increasingly recognized that body and mind interact in a bidirectional way. Movements, postures and inner bodily states are not only seen as resulting from mental processes and states, but they also have an effect on them, and physical sensations are recognized as an important source of information for arriving through a bottom-up process to the conceptual labeling of experienced emotion (Smith and Lane, 2015). This bidirectional interaction between body and mind is referred to as embodiment.

In the following paper, we describe an experience-oriented embodiment-based procedure that can be used to trigger, intensify and differentiate patients’ typical emotional processes within the framework of CBT. This approach makes it possible to initiate changes in emotional processes in a targeted manner, ensuring a higher level of commitment from the patient. We have developed and are using in our clinics this working format in which patients gain direct access to specific emotions through their bodies. This work includes, in addition to the more “classic” emotions dealt with in CBT, of fear and anger, other distinguishable emotions such as disgust, sadness, shame, guilt, etc. (Hauke, 2018b). These emotions can be explored, expressed and regulated using embodiment techniques so that ultimately goal realization can happen. The therapeutic dialogue between the patient and therapist, is then much more than verbal exchange: from the very first hour, we involve the entire body: movement, facial expressions, gestures, posture, voice and breath.

### 1.1 The embodiment perspective

According to current embodiment research, body and psyche are closely intertwined, i.e., the effects of body and psyche are reciprocal and mutual (Fuchs and Schlimme, 2009; Körner et al., 2015; Tschacher, 2018; Riskind et al., 2021). This bidirectionality has been demonstrated experimentally (Michalak et al., 2009; Adolph et al., 2021). For example, people who feel sad or depressed show typical physical non-verbal characteristics of sadness: their upper body is slumped and bent forward, and they move in patterns that are characteristically different from those of healthy controls. Conversely, if such posture, facial expression and movement patterns are unobtrusively induced in healthy controls, psychological characteristics of sadness appear (Flack, 2006; Michalak et al., 2009; Shafir et al., 2013). Furthermore, there are findings showing that certain breathing techniques also induce and intensify specific emotions (Bloch et al., 1991; Boiten et al., 1994; Philippot et al., 2002). For a review of the effects of movement and breathing patterns on emotions see (Shafir, 2015).

Tschacher also associates bidirectionality with the long-known “ideomotor effect,” according to which cognitive activities simultaneously activate corresponding muscular and visceral systems in the body (Tschacher, 2018). Bidirectional processes are conceptualised by him as circular interactions that are constantly active without us having to be aware of them. Embodiment theory thus states that pure abstract cognition alone does not exist; we are always unconsciously in an ideomotor simulation mode (Tschacher and Dauwalder, 1999).

A similar view is held by authors who advocate the “embodied simulation” approach. According to this approach, every form of cognition is linked to a simulation process in which the brain re-uses previous experiences of situations or internal or external stimuli similar to the current one, and simulates the interoceptive (i.e., input from the body to the brain representing the physiological state of the body, such as thermal, metabolic, hormonal etc.) and proprioceptive (i.e., input to the brain from the muscles and joints, which gives the brain information about posture and movement) information that entered the brain during that previous experience (Gallese, 2011; Hesslow, 2012; Barsalou, 2015; Barrett, 2017; Ross and Atkinson, 2020). According to Barret, the expected sensory feedback based on this simulation is then compared to the actual one, and this leads to the recognition, classification and naming of the current experience. As a result, emotions are embodied and facilitated by language (Barrett, 2017).

The embodied simulation of an emotionally charged situation can therefore also be seen as emotional activation. Imagining, remembering or visualizing during the therapy session, things one has seen, heard or felt before, activate the same processes in the brain as if one actually sees, hears or feels those things (Smith and Semin, 2004; Kosslyn et al., 2006; Niedenthal, 2007; Barsalou, 2009; Halberstadt et al., 2009). As will be described later, we are exploiting these effects of imagination and visualization, as well as the effects of movement and postures, in our suggested embodied approach to CBT.

For the purpose of emotional activation, i.e., the deliberate and artificial evocation of an emotion through imagination in therapy, classical CBT relies on various *in-vivo* exposure procedures (Craske et al., 2014) and on in-*sensu* exposure in particular. These exposure procedures are based on the approach of Lang, who postulated that the mental representation of an emotionally charged stimulus (e.g., a reference person experienced as difficult) activates an associative network of stored information that overlaps with the information that is activated when the stimulus is actually experienced in reality (e.g., when encountering the real person) (Lang, 1979). This formulation, reminds of the current embodiment simulation hypothesis.

### 1.2 Embodied emotion and emotion in CBT

The idea that the body affects emotions and feelings has been suggested already by James at the end of the 19th century. According to the James-Lange theory of emotion, bodily responses to external or internal stimuli are necessary for emotional experience, and therefore feelings are not the cause of autonomic nervous system activation and emotional behavior, but rather are the consequences of them. In the late 20th century this view, that emotions originate from the body, has been re-formulated in neurophysiological terms by the neuroscientist Antonio Damasio in his neurobiological hypothesis (Damasio, 1995), which is part of his famous somatic markers’ hypothesis (Damasio, 1996). According to Damasio, the current state of the body is conveyed to the brain through proprioception and interoception. These inputs from the body create in the brain unique neural activation patterns, whose purpose is to help us survive by causing us to behave in a way that will maintain our homeostasis. These neural activation patterns are our emotions.

In a recent review, the models that follow the path of James and Lange were referred to as “embodied theories of emotions” and they were contrasted with the so-called “cognitive theories of emotions” that form the theoretical framework of classical CBT (Smith and Lane, 2015). According to these authors, these two types of theories differ in what they regard as necessary and sufficient conditions for emotions. While according to the embodied theories, some kind of representation of bodily changes is required, for the cognitive theories the mandatory component is cognition, and physical changes such as arousal, visceral or musculoskeletal changes, will at best be seen as secondary by-products of cognitive appraisal. The body is neither necessary nor causally involved in constituting the appraisal. From this perspective, the cognitive theories of emotion show a quality of “disembodiment,” which is accordingly also predominantly evident in the way CBT works (Gjelsvik et al., 2018; Pietrzak et al., 2018).

Embodied theories of emotion, on the other hand, do not deny that emotions can be triggered by thoughts or judgments, or that appraisal is an important component of emotions, but they do not consider any of these processes to be mandatory for emotions to emerge (Smith and Lane, 2015). The authors suggest a new theory for emotions which combines both types of theories. Consciously perceiving and recognizing one’s own emotional state is understood in this suggested model as the result of complex, iterative bottom-up and top-down network interactions (Smith and Lane, 2015). Top-down based on previous experiences, expectations are created regarding the state of individual body parts, whole body patterns and the conceptual emotional meaning of these body patterns. These expectations are compared with the sensory input provided bottom-up by the body. Matching the expected with the real sensory input leads to the selection of appropriate actions. This idea echoes the embodied simulation theory described above.

### 1.3 Emotion regulation

The importance of emotion regulation in therapy stems from the fact that problems with emotion regulation (ER) are included in at least 75% of the diagnostic categories of mental disorders (Werner and Gross, 2010). In fact, it is well established that chronic deficits in emotion regulation occur in all major psychopathological categories.

An emotion regulation model that is close to cognitive emotion theories and CBT is that of Gross (1998). According to Gross, emotion regulation encompasses all processes that are aimed at controlling the spontaneous flow of emotions. It aims at the initiation of new or the modification of existing emotions, the accentuation, reduction, suppression or maintenance of emotional reactions (Gross and John, 2003). Emotion regulation serves to secure central needs, and supports the pursuit of specific goals and the global system of personality. Functioning emotion regulation is therefore essential for a person’s progress in developmental processes. Gross’s ER model (Gross, 1998) comprises five processes of regulation. Since emotions unfold over time, these emotion regulation strategies are often differentiated along the time axis of the emotion-generating process (although they can also run in parallel) (Gross, 2002): the first process is situation selection—we decide whether we want to approach or avoid a situation. The second: situation modification—changing the situation. The third: attention deployment, i.e., changing the focus of attention (e.g., distraction). The fourth: cognitive change (e.g., reappraisal), in which the meaning of the situation changes. The final process is response modulation—control over the emotional response (i.e., behavior, physiology, and experience).

The embodiment perspective encourages extensions of this classical model of ER. In this regard, we point out that the Smith & Lane’s emotion generation model described above also implies a model of embodied emotion regulation with several hierarchical neural and functional levels (Smith and Lane, 2015). Pollatos and Ferentzi (2018) contrast this model with Gross’s (1998) model and conclude that Gross’s cognitive approach corresponds to the explicit and goal-directed cognitive activities which only occur at the highest conceptual level of Smith & Lane’s model. This means that visceral and somatic mechanisms are essentially neglected in Gross’s model.

However, several studies showed that higher interoceptive accuracy (defined as the ability to accurately recognize signals from within the body) was associated with more intense feelings and higher activation of underlying brain structures or peripheral responses during emotional stimulation (Critchley and Garfinkel, 2017). Thus, interoception may particularly influence the experience of emotions when individuals are better attuned to their body signals. This means that the success of emotion regulation is influenced by the awareness of corresponding body signals, which demonstrates the integrative value of the embodied emotion regulation model by Smith and Lane (2015).

### 1.4 Emotional awareness

Perception and interpretation of bodily signals in the emotion generation process can be put within the framework of a cognitive-developmental theory of emotional awareness, which is inspired by Piaget’s theory of cognitive development (Lane and Schwartz, 1987). According to this theory, a person’s ability to perceive and recognize emotions in themselves and others is a cognitive ability that undergoes a development similar to that described by Piaget for cognition in general, whereby here we speak of “levels of emotional awareness.” When cognition is mentioned here, this also includes emotion, because emotion and cognition are seen as an inseparable unit, as is also the case by other authors of embodied cognition (e.g., Winkielman et al., 2015). These levels of emotional awareness are characterized by an increasingly higher degree of differentiation with regard to the processing of information from the body and the outside world (Table 1).

**Table 1:** Levels of emotional awareness according to Lane and Schwartz (1987).

∙Level 1: undifferentiated bodily sensations, e.g., sick, dizzy, sleepy
∙Level 2: perception of action tendencies or undifferentiated global affect (wants to hit a wall, wants to cry). Negative or positive valence (undifferentiated good or bad, stressful)
∙Level 3: specific concrete emotion (happy, sad, anxious, etc.)
∙Level 4: can name several emotions according to level 3 as well as emotional ambivalence
∙Level 5: perceive mixtures of emotions that differ for the self and other persons

A higher level of emotional awareness goes hand in hand with a greater ability to perceive the complexity in the experience of oneself and others. People with lower emotional awareness fail to interpret their body signals as feelings and instead only experience stress in a rather undifferentiated way on a physical level (Subic-Wrana et al., 2005, 2010), while the ability to precisely name and granulate the emotion associated with the increasing arousal is essential if an adequate response is to be developed (Erbas et al., 2022; Fugate and Wilson-Mendenhall, 2022). Thus, the high relevance of the concept shown in Table 1 for psychotherapy. In this context, the distinction between primary and secondary emotions is also of particular importance in therapeutic practice (Greenberg and Safran, 1987; Sulz, 1994; Fruzzetti et al., 2008). Primary emotions are normative, adaptive and also prototypical ways of reacting within a given context, for example, anger in the face of a barrier to satisfy a need. They show up quickly and seem almost reflexive. In the case of anger, for example, the body wants to go forward and attack. Primary emotions are primarily related to the person and their needs to be satisfied in the present moment. Secondary emotions represent a response to these primary emotions (Greenberg and Safran, 1987; Sulz, 2006; Fruzzetti et al., 2008). Particularly common secondary emotions are fear, shame, and guilt and they act as “stopper” of the primary emotions. They were learned in the past in order to spare the relationship with an important caregiver, because in childhood, when the fulfillment of needs was mainly dependent on caring adults, this was a skill essential for emotional survival. As will be described in the next section and illustrated through a case study, in our approach to CBT we use embodied techniques to help patients regulate their emotions by helping them to increase their emotional awareness and to differentiate between their primary and secondary emotions through increasing their interoceptive and proprioceptive awareness.

## 2 Using embodiment for emotion activation and regulation in CBT

**Figure 1:**
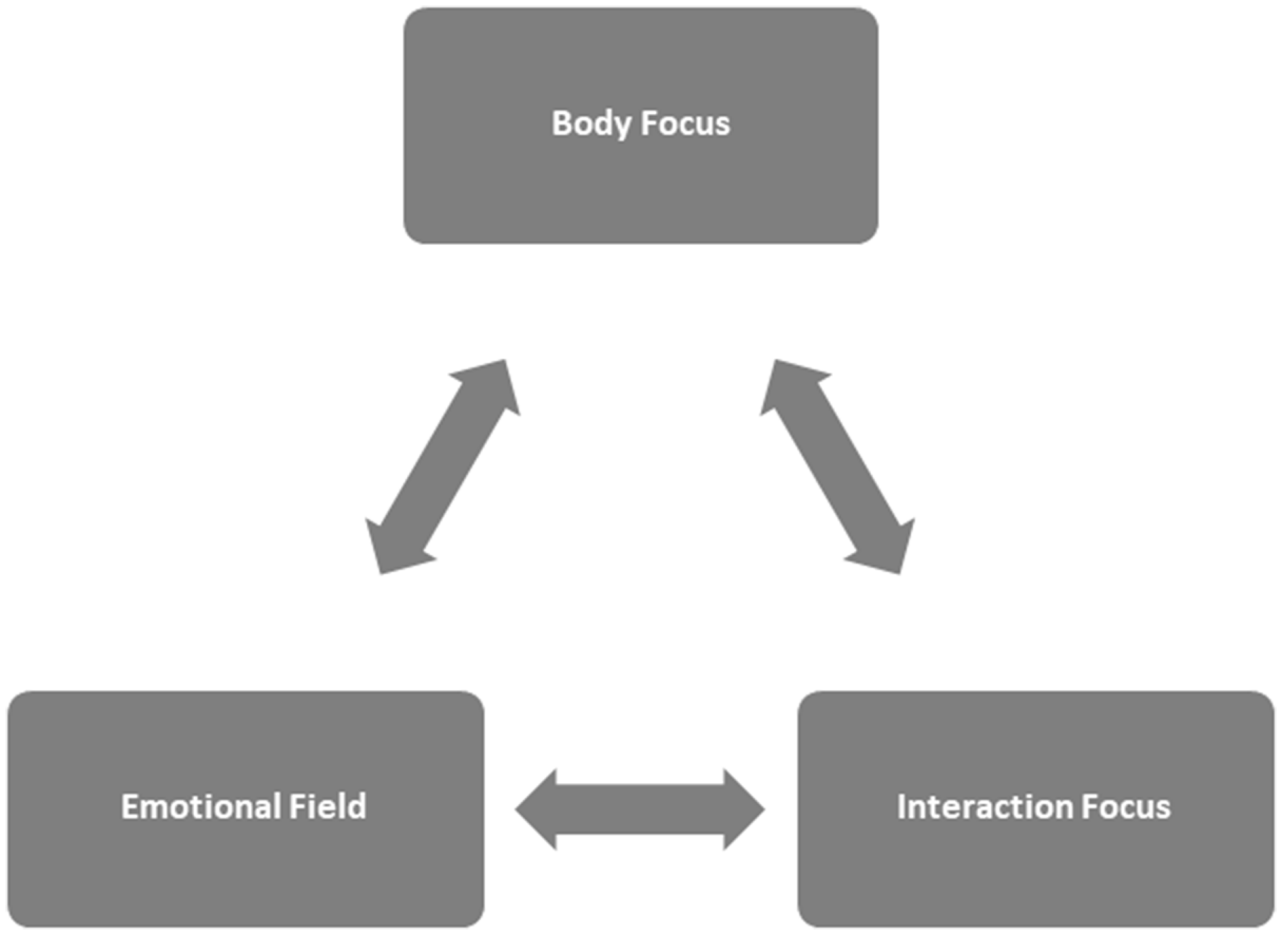
The three modules of the suggested embodied approach to CBT.

Our method of working presented below describes how to use in CBT the important insights of the concept “embodiment”. According to this concept, cognition and body sensations are inextricably linked, we take these dynamics into account by working in a circular manner on our three modules shown in Figure 1 body focus, Emotional field and Interaction focus.

These three modules will first be presented, with their central question in relation to the patient (Hauke and Lohr, 2019), and then be described in more depth. To clarify and demonstrate the proceedings and potential of our way of working in every step, we provide also a prototypical example case “Anne,” which was created based on clinical experience with several different patients. For a better understanding of the case, some of key biographical data and the results from a former CBT treatment are given below.

**Anne comes to therapy – again**

The 39-year-old patient Anne suffers from chronic depression. She is married, has a 16-year-old son and works as a preschool teacher. Anne was free of symptoms until six months ago. Anne describes herself as rather shy and less assertive. Her son Tom has started to rebel intensively. Lately he was brought home by the police after he had been fighting with peers. Anne is very concerned that her son is going down the wrong path. She tried to talk to her husband Marc about her concerns, but he only says that education is her responsibility. Complaining about the situation with Tom at her parent’s place, her father just accused Anne for not being tough enough. But her attempts to be more assertive with her son remained unsuccessful. During her arguments with him she starts crying early and loses. She sleeps very little and ruminates in bed, while waiting for Tom to come home from his parties. She feels more and more helpless and alone. Physically, she now suffers from severe stomach pain nearly every day. It's hard for her to keep her upper body upright with this pain. Therefore, she stopped her hobby horse riding.

**Anne’s biographical background**

She grew up in a strict home as the eldest sister to two younger brothers. She describes her father as a loud and dominant person, and her mother as a caring and calm individual, who mostly obeyed her father's commands. She met her husband Marc in high school. He does not like to talk about feelings, but is very reliable and hardworking. Anne feels therefore often alone with all of her worries and needs.

Anne behaves in daily life the way she learned it in the strict environment of her childhood, even though she knows from her previous therapy that she should take another path. Anne tries in vain to satisfy her need for affection and harmony with the same attempts of being easy to handle for others and with dutiful behavior. But with her teenage son she fails and exhaustion progresses.

**Anne’s former CBT treatment experience**

She experienced her first depressive episode, when her son went to first grade and she started working again. Anne often felt overwhelmed and worried that she would not be able to fulfill her duties in all parts of her life. Thus, she started her first CBT treatment which helped her a lot. As a result of the CBT she learned to be more organized and to do more self-care, and she started riding horses again. She also learned how to adapt her high household demands to her new role as a working mother and how to stop her automatic thoughts of self-criticism.

### 2.1 Module 1—body focus and safety

This module deals with the question:

"How can a patient feel calm and secure in his/her body, and turn to it calmly so that it can effectively support them in achieving their goals?"

Emotion regulation begins with the body. Stressful developmental conditions in the family of origin, verbal and physical experiences of abuse and violence, experiences of massive devaluation in peer groups, etc. prevent the body from being experienced as a safe shelter. The establishment of the body focus and safety are essential for addressing patients’ problems in a truly effective manner, because only those patients who feel safe can attend to their bodies (Podolan and Gelo, 2023), and only those who can observe themselves, will become aware of their emotions and needs (Beitman and Soth, 2006).

#### 2.1.1 The body as a safe shelter

The usually so self-evident feeling of having one’s own body—the so-called bodily self-awareness—presupposes that one feels that his body belongs to oneself and is anchored in a certain place in space (Iachini et al., 2014). This basic feeling arises from the processing of information within the so-called “**peripersonal space**,” a zone that extends around the body roughly within arm’s reach (Bogdanova et al., 2021).

To ensure appropriate and safe interaction with our physical and social environment, our brain makes a clear distinction with respect to spatial conditions: Objects present in, or moving into, the peripersonal space, receive special attention because it is in this zone that most interactions with the environment take place. The peripersonal space is also considered a safety zone that is important not only for the regulation of ordinary interactions, but also for rapid responses when dealing with stress in social situations (Iacoboni and Dapretto, 2006). Thus, objects that enter this zone might activate motor programs for goal-directed actions such as avoidance or defensive movements. Information processing regarding the peripersonal space also enables predictions of negative consequences during physical contact, e.g., when one must protect oneself from unwanted intrusion. This intrusion can act like an invasion that immediately spikes physiological measures of stress (Evans and Wener, 2007). An example of this would be an assault, causing trauma.

Therefore, it is important to give patients the opportunity to, not only experience security within the therapeutic alliance, but also to physically experience security in their body and to perceive it sensually. As we will see in the example case, the simplest and clearer way to do so, is first to determine a safety zone and physically defining it in the space of the room (e.g., with the help of a rope). As a second step, practicing defensive body reactions and verbal signals to protect this zone is another very helpful way to support patients to establish and strengthen their body focus. For more detailed information on the methods just mentioned, please read (Hauke and Lohr, 2020).

#### 2.1.2 The body as a source of information

People interact with their environment through their bodies. They touch and are being touched, and they see, hear or smell objects, people and events in their environment. The sensory perception of these stimuli immediately triggers an emotional neurobiochemical response in the body. This response indicates the extent to which the situation is relevant to the person and it makes itself felt through bodily sensations such as abdominal pressure, pain, blushing, changes in breathing pattern, nausea, and more. This neurophysiological barometer informs whether an object or situation is supportive or harmful, rewarding or threatening, and motivates approach or avoidance behavior. The ability to consciously perceive such bodily sensations not only influences the experience of a particular emotion but is crucial for a successful use of emotion regulation strategies (Füstös et al., 2013; Pollatos and Ferentzi, 2018). Thus, it is important to take the body seriously as a source of information and specific body signals as essential information for behavioral control in a wide variety of situations. Noticing and recognizing rising body sensations and impulses to act, require body focus and attention to what one feels or senses, which necessitates some practice.

Body scanning, mindfulness and mirror exercises have proven themselves as effective methods for achieving such bodily awareness (Weineck, 2018). Helping the patient to find simple verbal descriptions for the body events, in elementary, straightforward “feeling language,” instead of creating hypotheses and concepts, is also very useful. Examples of such simple bodily descriptions are:

- Feeling heavy hearted
- Feeling butterflies in the stomach
- Having a lump in one’s throat

Becoming aware of one’s emotions based on bodily sensations is a bottom-up process. These “messages” of the body are then put into simple words by the patient together with the therapist, and, if necessary, put into further contexts in a top-down oriented way of working. Both working principles are illustrated by Weineck (2018).

**Anne’s body focus work**

When Anne started to tell her new therapist about her current situation at home, she said: “I know I should feel more anger and act more tough with my grown-up son! My last therapist told me so already. But it is hard to describe. Anger for me is just a logical thought, but I have never really felt it in my body. In childhood I saw my brothers fighting furiously and afterwards laughing together. That was a very strange world for me, which I still cannot understand. Being physically angry just doesn't feel like me.”

The therapist explains to Anne that, in fact, she may not yet have found physical access to her anger. In order to find this access, it is important that Anne knows her body and its reactions very well. However, the current stress in Anne's life makes this very difficult, because she is often way too distracted, for example, when waiting for her son to come home at night. In order for Anne to be able to devote herself to observing her body reactions in peace and quiet, while doing mindfulness exercises for example, she first needs to feel a sense of security again. During the therapy session, Anne is given the task of defining a space around herself with a rope, in a size which she finds comfortable. At first, Anne finds the task unusual, but soon rejoices in having space all to herself. Standing inside this space she feels her breathing becoming easier, a smile forming on her face and her stomach loosening. Anne is surprised by the rapid changes in her body sensations, when she makes her space smaller: her chest gets tight and her stomach tense. She knows this feeling too well from her everyday life. Anne gets” homework” from her therapist: to look for a place at home where she can claim her space and mark it on the floor. She should visit this space every day and practice her mindfulness exercises there for 15min.

Anne opts for the guest room on the top floor of her house. There are only a few pieces of furniture there and she is undisturbed even when her husband and son Tom are at home.

### 2.2 Module 2—the emotional field: getting out of the diffuse experience of stress!

The problem-solving process begins with emotion-activation work because emotions provide navigation and vitality in a quick and accurate manner. The emotional activation and processing take place in the so-called **Emotional Field** (Hauke and Dall’Occhio, 2013). This work deals with the question:

"What is the network of experiences and emotions that repeatedly steers the patient in problematic directions?”

Patients come to therapy to solve problematic situations. Such situations offer possibilities for exploring very valid, favorable, or unfavorable strategies of emotion regulation. For a deeper understanding of the patient’s problems, it is crucial for both the therapist and patient that the explored emotion regulation strategies are not only talked about, but that they are directly experienced and demonstrated. Experiencing emotion regulation strategies increases both quality and speed of emotional processing. The Emotional Field does justice to the fact that in an interaction situation (e.g., in a marital conflict) patients experience usually more than one emotion. Several emotions are usually unfolded in parallel or one after the other. Experiencing the emotions in the Emotional Field helps to lead the patient from the initially diffuse experience of stress to distinguishable emotions.

#### 2.2.1 Structure and composition

**Figure 2:**
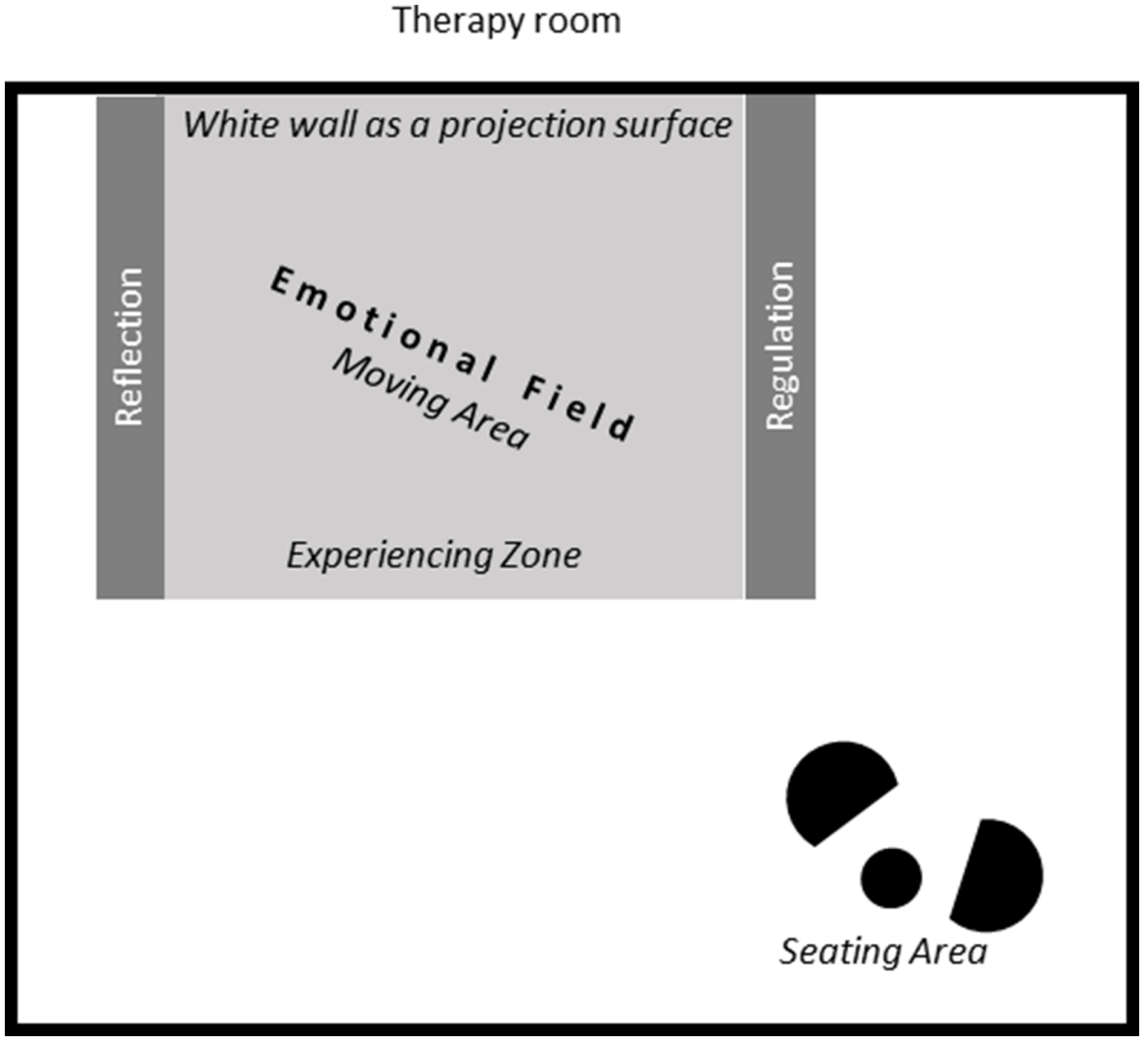
A potential arrangement of the therapy room for applying the suggested embodied approach to CBT.

The patient’s experience in daily life is brought into the therapy room using the “Emotional Field”—a circumscribed empty space, separated from the seating area and the rest of the room (represented as the gray colored areas in Figure 2), which is marked on the floor as an area to move in Figure 2 shows a possible arrangement.

This space provides room for several different spatial positions, which are marked by lines, using removable tape or ropes, and later on, with labeled cards. The lines separate the experiencing zone from the two lateral areas, where the exact location of the expert position-dedicated for reflection, and of the neutral position-dedicated for emotion regulation, can be chosen by the patient. By dedicating different places in the room to different parts of the therapeutic process, the bottom-up emotional work which is mostly about perception and sensing, is clearly separated for the patient from the top-down work of reflection and regulation. Changing position in space helps the patient to make the distinction between feeling his body and talking about insights.

Thus, the patient is supported in distinguishing between experiencing, analyzing, and regulating emotions, which are all different kind of useful practices that take place in the successful process of emotion regulation. These practices are benefitted by using the body and its changes of positions in space.

**Figure 3:**
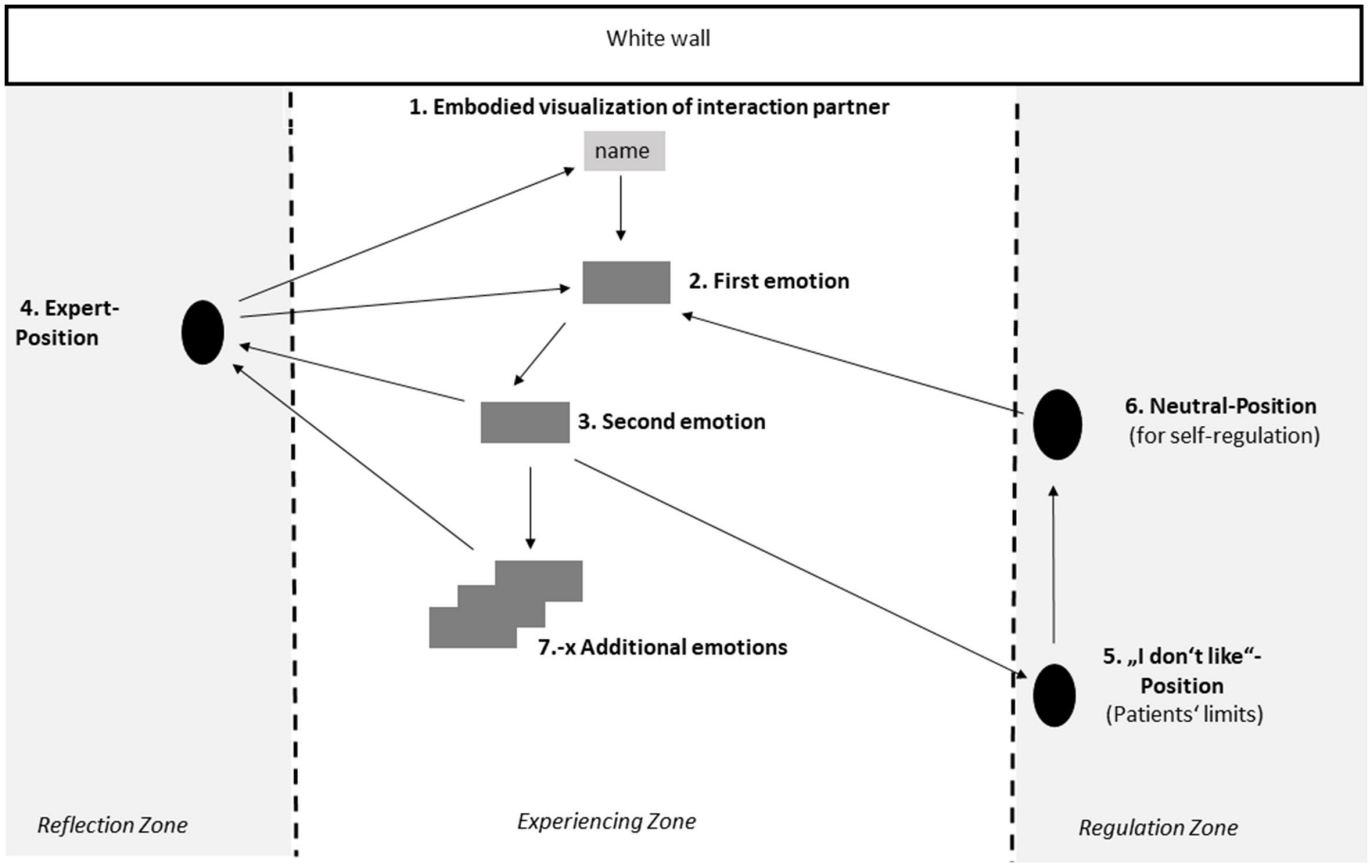
The therapeutic process which takes place in the emotional field and its various spatial positions.

Figure 3 gives a more detailed insight into the composition and therapeutic process which takes place in the Emotional Field and its various spatial positions as described in details below.

The patient can concentrate on working in one spatial position, but also has all other positions in view, so that s/he can move to work in them when necessary. In the beginning of the therapeutic process, cards labeled with different emotions (shown as grey rectangles in the figure) are placed on the floor by the patient to spatially mark the different positions, and s/he can move their location within the experiencing zone as the therapeutic process unfolds. Throughout the therapeutic process, the patient moves freely, and switches between the different positions in the room. The main embodied emotional work takes place in the experiencing zone, starting from the confrontation with the interaction partner and alternating with the need to analyze and regulate. The therapist follows the patient as s/he moves from one position to the next, stands to their side or behind them, and makes suggestions for position changes among the following positions:

##### 2.2.1.1 Position 1 – starting with a vivid visualization and embodiment (through mimicry) of the problematic person

The emotional work at the Emotional Field starts with an embodied visualization and enactment of a problematic situation with a relevant interaction partner (e.g., family member or a colleague). Designation or name of the relevant person is written down on a card. This card is placed in front of the white wall, and the patient visualizes the person in the problematic situation standing at that point and interact with him/her as vividly as possible—including all possible senses. The patient then puts him/herself in the shoes of his/her interaction partner and imitates their typical posture, gesture, tone of voice and facial expressions. By doing so, the interaction partner’s emotional impact on the patient becomes more perceptible for the patient and therapist.

##### 2.2.1.2 Position 2 – experience the emerging of the first emotion

Back in his/her own shoes the confrontation with the relevant interaction partner usually leads to a rapid emotional reaction, expressed through physical sensations and impulses. Patients often use expressions such as “Now my chest is getting tight!,” “I feel heat in my head!” or “I would like to sink into the floor!.” The patient uses these bodily sensations as clues to the emerged emotion.

##### 2.2.1.3 Position 3 – experience the emerging of the second emotion

If patients are guided to deepen the experience of the first emotion with the help of embodiment techniques (see section 2.2.2), another emotion usually appears after about 1–2 min. It announces itself by a change in the body sensations. This “tipping point” between the emotions is usually accompanied by spontaneous expressions, such as “Now I want to straighten up!,” “I feel the energy returning to my body!,” or the patient suddenly starts to cry.

##### 2.2.1.4 Position 4 – talking at the expert position

The expert position is placed by the patient at the edge of the Emotional Field and serves for both patient and therapist as a place for reflection. Both are experts: the patient for his/her life, and the therapist for psychological contexts. Here, what has been experienced in positions 2 and 3 in a bottom-up process, is now discussed and classified in a top-down process. At this position the cognitive therapeutic work is done, including the referencing of the experienced bodily and emotional responses to the patient’s biography and the formulation of the patient’s reaction chain based on the concept of primary and secondary emotions (see section 2.2.3).

##### 2.2.1.5 Position 5 – exit option at the “I do not like”-position

In such an intense therapy process, security and control must be guaranteed for the patient. This includes that resistances are taken seriously and are given their own position, the so called “I-do not-like”-Position. The patient can switch to this position, whenever the feeling arises that s/he wants to end the exercise—for whatever reason. Sometimes, after talking about the reason with the therapist, a continuation of the therapeutic process is possible.

##### 2.2.1.6 Position 6 – self-regulation on the neutral-position

The patient can choose this position, whenever s/he feels overwhelmed from the intensity of an emotional episode. In this position, the therapist leads – a previously practiced and therefore well-known – emotional neutralization exercise, which involves the whole body, e.g., a simple physical exercise like the one Bloch (2006) recommended.

##### 2.2.1.7 Position 7-x—experiencing the emerging of additional emotions

Problematic situations are usually linked to a network of emotions. Usually, several other emotions emerge beside primary and secondary emotions. These new emerging emotions are then also deepened, named, and experienced with the help of our embodiment techniques (see 2.2.2) and are used in the further therapeutic process.

The process of experiencing the emotions and reflecting is done at different positions in the Emotional Field (see Figure 3). At first, the work concentrates on the immediate perception, feeling and simple description of bodily reactions that arise in response to the experience. This approach to physical sensations corresponds to the bottom-up method described earlier (see section 2.1.2). It is necessary to provide access to pre-linguistic content. Once the emerging emotion is clear, the work in the expert position switches to top-down analysis of the situation and the emotional response to it in relation to one’s biographical history. The top-down oriented work by both patient and therapist together, takes place at the “Expert-Position.” Work at this position supports mentalizing: What was previously experienced in the present moment is not only visited, but also classified, analyzed, differentiated, and questioned. This stimulates a more precise understanding of the emotional messages and the patient’s own behavior. Through this process, the patient becomes the “expert” for his or her experience.

**Anne is entering her first Emotional Field**

Anne decided to work on a problematic situation with her little supportive husband. The argument arose when Anne wanted to explain to him that she needs his help with Tom and that she wants him to back her up. In order to ensure an intensive emotional activation for Anne, she is first asked by the therapist to portray her husband as vividly as possible. Therefore, Anne first places the card with her husband's first name in front of the wall and stands on the card with her back to the wall as a sign that she is now slipping into his shoes. Guided by her therapist, Anne chooses a typical body posture of her husband, a phrase that he frequently says, and his corresponding facial expressions, so that Anne’s husband becomes tangible as the central reference person in the room. Anne then positions herself in front of the wall that shows her "visualized" husband. Anne chooses a shorter distance from the wall and a crouched position, because she has always felt a bit inferior to him, because of his high self-esteem. Supported by the therapist, Anne now begins to describe what she feels in her body. Anne reports a tightness in her chest that makes her breathing shallow. In addition, she feels an impulse to make herself even smaller. Anne should now give in to this impulse. She crouches down and condense her body very tightly, until she can hardly breathe and her stomach starts to hurt. Anne should now wait for the next body impulse and give in to it. After a short time, she reports a heat rising in her head. Anne feels an urgent desire to get up. However, when Anne stands up in front of her husband again, the heat immediately disappears. Anne wants to make herself small again. At this point, the therapist asks Anne to move to the so-called expert position a reflection point in the room located away from the experiential zone in front of the wall. Here Anne and her therapist try to give a name to the different emotional states she has just experienced. The first emotion that emerges is very familiar to Anne. It is fear, which takes away her breath and helps her to hide. Anne is familiar with the strange heat, but she cannot think of a more concrete name for it. The therapist writes down the two terms ‘fear’ and ‘heat’ each on a different card. Anne places both cards in front of her visualized husband. After this short conversation, the therapist asks Anne if she feels ready to go into the experiential zone again and find out more about this heat. Anne agrees. Both Anne and the therapist start again with the confrontation of Anne with her husband in front of the wall. As in the first run, Anne's fear shows up first, which—if intensified—again creates an impulse to stand up and heat in the head. The therapist asks Anne to reinforce these body signals as well. Anne now also feels a strong desire to move closer to her husband and reports an impulse to yell at him. She yells, "This is your son, too! Do something!" Now Anne feels significantly taller. To reinforce this feeling, her therapist gives her a wooden stool to stand on. From here she can now look down on her husband, but at that moment all courage leaves Anne and she immediately wants to make herself small again and to back away. The therapist lets Anne follow her body impulses and then discusses her experience again at the expert position.

#### 2.2.2 Embodiment techniques using movements characteristics

Information processing by embodied cognition allows the body to become an ally in the Emotional Field (Hauke et al., 2016; Hauke and Lohr, 2020). Embodiment techniques can include the use of specific breathing patterns, facial expressions, direction of gaze, postures, gestures and movements that include specific movement characteristics. Starting with the work of Bloch (1989), Bloch et al. (1991), and Bloch (2006), who developed specific patterns to evoke emotion through posture, facial expressions, gestures, and breathing patterns, further studies used specific movements and postures (e.g., Duclos and Laird, 2001; Carney et al., 2010; Shafir et al., 2013). The question of whether any of these patterns are hardwired in the brain (e.g., Cowen and Keltner, 2017, Saarimäki et al., 2018, Nummenmaa, 2022) or can be learned in context and facilitated by language (Barrett et al., 2007; Cristaldi et al., 2024) is an ongoing debate.

A particularly promising innovative approach in evoking emotions is the use of movement characteristics defined by Laban Movement Analysis (Shafir et al., 2016), since, like hardly any other approach, it enables each individual to evoke the emotion using their own personal motor repertoire, rather than using very specific movements, which might be physically difficult or unnatural for them to execute. Laban Movement Analysis, originally conceived by Rudolf Laban, is a comprehensive, well-established and widely accepted movement-analysis method, which provides systematic language for describing, documenting and interpreting qualitative and quantitative aspects of movement, by defining various types of motor components (Bartenieff and Lewis, 2002; Studd and Cox, 2013; Fernandez, 2015).

Table 2 shows examples of movement characteristics which when moved can induce or enhance specific emotion. These movement characteristics which are based on motor components from Laban Movement Analysis (written below with capital letters), are described in the table in language that can be understood and applied by everybody.

**Table 2:** Examples of movement characteristics which when moved, induce specific emotions.

Emotion	Movement characteristics to evoke and deepen this emotion
Happiness	Jumping, Rhythmic movements such as tapping, skipping, or dancing, moving (the arms and head) Upward, Rising (the chest area), Light movements, Spreading [opening to the side the arms, legs and torso (e.g., widening the chest and shoulders)], Rotating (either the entire body as a whole, i.e., turning around one self like in a Sufi dance, or the upper body, i.e., twisting)
Sadness	Head drops down, Sinking of the chest area, Passive Weight, i.e., feeling very heavy, moving Downward towards the floor (e.g., dropping from a standing position to the knees), Stillness (i.e., not moving at all), Enclosing the body, Near Reach Kinesphere (i.e., moving small movements within a small peripersonal space), touching the face or upper body (neck, chest, shoulders), slow movements.
Anger	Expand the body and make it big, Advance the chest area forward, Strong, Direct (i.e., towards a specific target), and Sudden movements with Bound flow (i.e., in a very held and controlled way)
Anxiety	Condensing and Enclosing the body, Retreating backwards in the chest area, Moving Backward and Downward in the general space, Bound Flow (i.e., moving in a very held and controlled way), Near Reach Kinesphere (i.e., moving within a small peripersonal space)
Disgust	Condensing the body (i.e., making it small and narrow), Gestures of bringing the hand to the stomach/center of the body, Twist the upper body away from the disgusting object, move Backward in space, move as if one is throwing up (enact vomiting).
Pride	Direct and Strong Upward movements of the arms, Spreading the body (e.g., wide stance, arms to the sides, wide and open chest), Rising the chest area
Shame	Sinking down and Retreating backward the chest area while the head is rotating sideways and tilted down, looking down, or when moving forward—Head Rotate to look sideways, covering the face with both hands, moving with Bound Flow

The advantage of using movement characteristics as opposed to specific movements is the ability to personalize the required movements for evoking specific emotion, adjusting them to each patient based on their personal movement vocabulary, their motor coordination and their range of motion, as well as their natural whole-body emotional expressions. This way, the patient does not have to move a specific movement which s/he might not feel comfortable with, or simply cannot physically perform, but can move any movement as long as it includes one or some of the movement characteristics associated with that emotion. For example: to evoke anger, the patient might use the combined movement characteristics: Strong, Direct and Sudden by kicking or stomping with the leg, or by punching, hitting the table strongly with the palm, or slamming a door, with the arm(s). All the above-described movements are done with the movement characteristics (Laban motor components) of Strong, Direct and Sudden. People who have physical or emotional difficulty to move strongly and directly (e.g., people who prefer avoiding confrontations), can advance in the room pushing their chest forward while expanding their body and raising their arms to the sides, or just make strong fists and push their chest forward slowly and in a very controlled way (Bound flow) while standing in place, if they have difficulty with and are usually trying to hide or avoid anger expression.

The induction of emotions through movement, though, has to be done gradually, if we want it to feel natural and to really affect the emotional state. A depressed person cannot just start dancing and feel happy, because such abrupt change in his movement will feel awkward and artificial, if at all possible. The movement characteristics which evoke the desired emotion should be added to the patient’s movement gradually, one by one. The theory of Laban Movement Analysis suggests principles that can be used to support such gradual change in one’s movement pattern. These principles and how to work with them are described in details in Tsachor and Shafir (2017).

Lastly, it should also be noted that getting rid of, and stop using a motor component that is associated with a specific emotion, can weaken the feeling of that emotion. For example, if a sad patient always sinks in his chest and constantly looks at the floor with his head dropping downward, standing erect and looking forward may already reduce some of his sad feelings.

**Anne learns to explore and deepen her anger**

When the therapist realizes how difficult it is for Anne to vent her anger, he suggests another exercise based on the movement characteristics. Anne is given the task of going completely into her fear and making herself as small as possible. Step by step, she is now being guided by her therapist to first give up the movement characteristics of fear, starting with the condensed and crouched posture. Anne is asked to lift her head, slowly sit up and look around. Then she is asked to stand up and carefully take her first steps. Now the therapist guides her to gradually incorporate into her movement the movement characteristics of anger. She begins by moving forward more purposefully and speeding up a little. Later on, Anne expands her body. Anne is repeatedly asked about her feelings and how they change during the movement. The aim of this exercise is to let Anne consciously feel the tipping point between her fear and her anger, but above all, to physically feel her anger very consciously and carefully. Anne reports after the exercise: "It was amazing. I felt my fear so well in the condensed posture and after a few seconds my stomach started hurting like hell. It only went away when I got up and began to breathe freely again. I wouldn't have associated my purposeful movement with anger at all, but it helped me a lot to focus and feel kind of more confident. But it wasn't until I pushed my chest forward and expanded my arms, that I really felt angry. I immediately started feeling hot and powerful and many swearing words that I would like to yell at my husband, came into my mind.”

These two emotions – fear and shame—being dominant in Anne’s body and mind, led her to patterns of submissive behavior. Anne has already understood mentally that her submissive behavior would not help to set boundaries to her son or win arguments with her husband, but she was unable to act otherwise, because the physical and emotional basis of anger were missing in her body. Being guided step by step to experience the tipping points between primary and secondary emotions with the help of the movement characteristics, helped Anne to develop a strong physical connection to her anger before using it in interaction.

After deepening the connection to her anger, Anne is now able to feel the power that is connected with the anger and this awoke her interest. Now she is ready to learn, how to dose her anger appropriately.

In the way we work in the Emotional Field, we use the movement characteristics in particular, when it comes to emotions that the patient is hardly aware of, or has not yet fully developed to use them appropriately in his/her life. In addition, the gradual development of emotional expression also allows the patient’s body awareness to be improved. S/he is thus able to consciously try out every physical component of his/her emotional expression and trace changes in his/her emotional experience. This enables a very targeted and individual work with emotions.

#### 2.2.3 Evaluating experience in the emotional field – understanding the reaction-chain

Once emotions are activated by imagination via embodied simulation, they can entail different reactions within the person. These reactions have different causes and therefore also different intentions. The unresolved dilemma of different impulses to act, triggered by different emotions at the same time, creates the overwhelming moments of stress that the patient experiences (Renna et al., 2020). Therefore, as explained in section 1.4, it is very helpful in the therapeutic work, to distinguish between primary and secondary emotions (Greenberg and Safran, 1987; Sulz, 1994; Fruzzetti et al., 2008).

For most patients, secondary emotions have turned into automatic responses and they now hide the primary emotion beyond recognition and consciousness. Hence, in the Emotional Field the well-known secondary emotion emerges first and the primary emotion usually shows up afterwards. If the impulse of the primary emotions had to be suppressed strongly in the family of origin, a bouquet of secondary emotions often arises to cover it “watertight.” The spontaneous, primary emotion is thwarted, and another emotion is displayed instead—a simple and effective form of emotion regulation. These additional emotions can also be discovered in the Emotional Field and can be used in the further therapeutic process.

**Figure 4:**
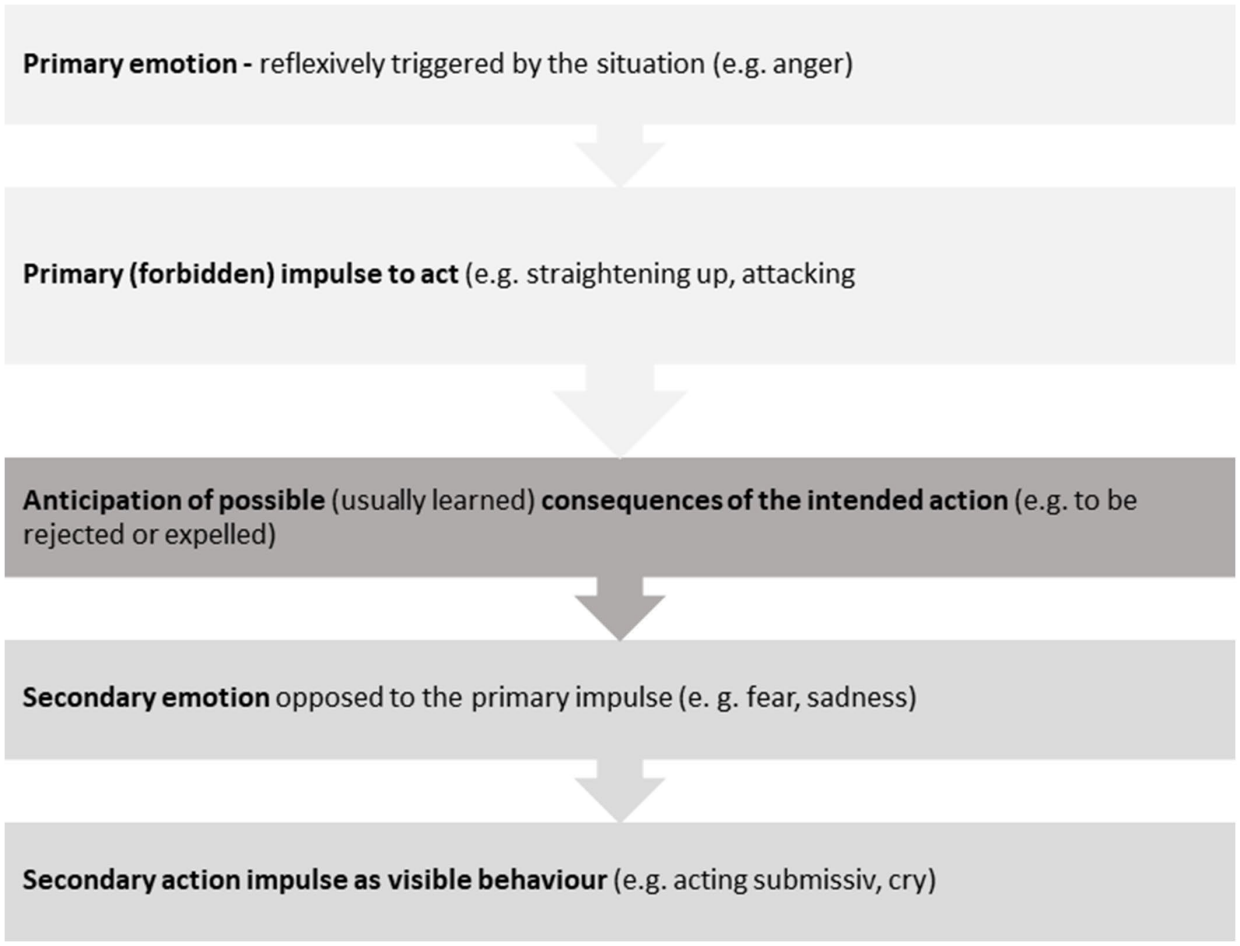
General structure of a reaction chain.

Since the emotional processes happens so quickly, most patients cannot differentiate between the primary and secondary emotions anymore without therapeutic support. Methods such as the so-called reaction chain (Sulz, 2006; Hauke, 2018a) were developed to make the connection between primary and secondary emotions cognitively clear to patients. Figure 4 shows the general structure of a reaction chain, as it can be used for work with patients.

Even if this reaction-chain pattern is logical for the patient and cognitively easy to understand, while analyzing it with the therapist, acceptance and the motivation to change behavior in daily life is often much more difficult. However, the situation is different once the patient has physically experienced the tipping point between primary and secondary emotions, as described below in the case “Anne.”

**Anne’s reaction chain**

Working with the Emotional Field allows Anne to consciously and slowly experience the emotional processes that otherwise take place at high speed in real conflict situations. Anne is now able again to physically perceive the signals of her primary emotion: anger. This is an experience that wasn't possible for Anne for a long time, because she had learned very early in life to block her anger with her secondary emotions fear and shame. The body impulses are more physically noticeable for Anne and make it easier for her to understand what happens to her during conflicts. The so-called reaction chain becomes apparent.

Several times, Anne goes back to her experience, and thus shame gradually becomes apparent as an additional emotion associated with this situation. By working in the Emotional Field, Anne understands better why it is so difficult for her to stand up for her needs in everyday life: because not only her fear but also shame slows down her feeling and expression of anger. She has a strong duo of stopper feelings (secondary emotions), which ensure that she doesn’t risk any relevant relationship.

After repeatedly experiencing these tipping points, which can be deepened and tailored to the patient's possibilities with the help of the previously described movement characteristics, the cognitive classification is immediately accompanied by commitment and long-lasting insights. This creates a solid basis for further work on acceptance and behavior change, from which all well-known methods of CBT, such as symptom therapy, benefit.

Experiential understanding is important, but usually not enough to achieve sufficient problem solving and robust behavioral changes. Therefore, new behavior is encouraged to be planned and tried out during the third module.

### 2.3 Module 3-interaction focus: satisfying needs around others!

In the **interaction focus**, the insights gained while establishing the Emotional Field are applied to various problematic situations and adapted to different framework conditions. For example, goal-directed behavior can depend on the extent to which one succeeds in matching the meaning and power of one’s own emotions to personal characteristics of the counterpart. This work is guided by the question:

"How can patients achieve goals while feeling ("agentic") self-efficacy?"

For many patients, the quality of their life depends on successful goal achievement (Kuhl et al., 2015). Developing and achieving one’s own goals is only possible if energy is permanently available. However, this energy is only available, if survival is ensured and one’s own needs are reliably met over the long term. Needs can only be satisfied if sufficient physical resources are available to react to the conceptualised emotion and carry out the associated action impulse (Shaffer et al., 2022). No matter what the type of need is, meeting it almost always involves interacting with others. The body plays a central role here, because it helps us to regulate the proximity to other people and to exert our influence on others.

A successful interaction with others requires one to be able to control his/her emotion in such a way that it reaches the other person in a favorable way.

To do this, s/he must consider what type of person is the individual with whom s/he interacts. Is it a very dominant person, a good sparring partner or rather a person who avoids conflict? Only if the self-regulation in emotional self-focus is already working to some extent, s/he can face the demands that come from the situation and from his/her counterpart person. Mastery of emotion regulation is ultimately demonstrated by the fact that competent handling of emotions leads to the improvement of relationships and, ideally, to the deepening and broadening of intimacy and closeness.

Successfully regulated interaction also requires clarity about what one wants to trigger in an interaction partner. Does one want to trigger care, provide care, form an equal alliance, assume a certain position of power, or perhaps enter a sexual relationship? Such questions can be quickly brought to the point by representation in space. For example, if a person is perceived as strong and attractive, he or she is automatically positioned “up,” immediately implying an interaction between “up” and “down”: the protective giver (up, tall) provides security for the receiver positioned further down (short). Such a relationship can be easily represented spatially and this representation helps to make abstract concepts such as dependency and subordination tangible. A chair can help create vertical distance in each situation. When a patient stands in front of the chair or on the chair, s/he grasps and experiences the status differences with impressive intensity. This approach takes up the concept of power (above/below), physically creates the psychological meaning and thus supports the simulation of the abstract concept of power (Carney et al., 2010; Cuddy et al., 2018). It is not only the positioning of the body that promotes the understanding of a conceptual metaphor. Likewise, the experience of emotional proximity or distance can be quickly realized in space. Experimenting with the horizontal spatial dimension sheds light on certain aspects of the psychological proximity. By varying these spatial parameters, emotionally charged issues can be quickly made perceptible and visible (Frank, 1997). Such effective embodiment techniques are guided by empirical findings (Schubert et al., 2009; Davis et al., 2011), but will not be elaborated further here.

**Working in the interaction focus with Anne**

Now, that Anne has solid experience with her own anger and she feels comfortable feeling it in her body, she is well prepared to start working with the interaction focus. For Anne her goal is clear: She wants to use her anger to interact differently with her husband Marc. She says: “I don't want to yell at Marc out of anger. I know him, he would just leave the room. I want to make it clear to him that I don't understand his attitude and that I really need his help with our son."

The therapist invites Anne to imagine her husband again in front of the white wall. He asks her to show him how (psychologically) big or small she feels towards her husband while expressing her needs as clearly as just now. She marks the psychological size she has chosen with a post-it on the wall and says: “Back in the Emotional Field, I felt much smaller than Marc. Today I feel much taller than Marc. That is a strange and unfamiliar feeling for me. It is confusing.”

The therapist asks Anne to get up on a chair, to intensify her feeling of being taller. Feeling her own body reactions while looking at the much smaller height of her husband at the wall, Anne said: "Being above him makes me feel very safe. I'm not afraid of any of his reactions anymore. But somehow it doesn't fit my need to ask him for support.” The therapist asks Anne to find a position that better suits her need. Anne is a bit confused at first and says: “On the one hand asking for support is something that makes me feel smaller, because I feel, I can't do it on my own. On the other hand, I can clearly feel anger in my chest, because I know I deserve Marc's support here.” Her therapist encourages Anne to choose a size ratio, which she has not tried before. Anne chooses the same size ratio and says: “I feel so well, being on eye level with Marc. But it is more difficult than expected to keep the feeling over time in my body. After a couple of seconds, I feel a well-known impulse in my shoulders to make myself smaller and condense. If I do so, my anger immediately counteracts and I want to expand my arms and make myself much taller. I have never been able to feel all of my body impulses so precisely.” Anne realizes for the first time how much her body awareness has increased in the last months. Her therapist uses her new body awareness to find the fitting intensity of emotion for her conversation with Marc. To do so, he works again with the movement characteristics of anger. For Anne pushing her chest forward, was one of the best working movements to increase her anger feeling. She starts to produce a low-intensity anger by pushing her chest slightly forward and at the same time she resists bringing her shoulders down. She is practicing all of these little movements by looking at her husband on the wall at eye level. After Anne feels secure with her body posture and feels her own assertiveness in her chest, the next step comes.

Anne and her therapist now discuss which arguments she would like to use in the conversation with Marc. Once these are found, Anne combines her assertive body posture with her arguments and tries to find the fitting tone and volume of her voice. Anne says after she finished the exercise: “In my last therapy, I practiced a lot what I wanted to say to Marc. It often worked quite well during the therapy session, but when I got home, I lost courage or my attempts failed miserably. I felt like a bad actress who didn't play convincingly. But now it's different. I have my body as an ally and have learned to develop the fitting body posture first. My words than easily follow my body.” After her first attempt to talk to Marc at home, Anne says in her next therapy session: “I managed to get all my points on the table. I remained so calm that Marc stayed in the room and listened. He said he needs to think about my arguments. And what is most important for me: I feel confident to bring up the topic again, if Marc is not getting back to me in the next week.” With the help of embodiment techniques, Anne was able to recognize her body's signals and use them according to the situation. Her knowledge of how her body expresses emotions and how to use this knowledge to get her needs successfully fulfilled by others, allowed Anne to feel self-effective again: an important factor for her relapse prevention and her successful therapy.

## 3 Discussion and conclusion

As described in the introduction the neuroscience-based concept of “embodied cognition or embodiment” represents the view that cognitive processes are not possible without the direct participation of the body. Acting in any situation, the body has direct influence on cognitive, motivational and emotional processes, through its posture, movement, facial expressions, etc. Thus, embodied techniques can be used in CBT practice both as a tool and as an important source of information: one can consciously and willfully activate and/or regulate his/her emotions by using specific movement patterns. In addition, attending to one’s body and sensory experience, can help patients to identify their emotions more accurately, to perceive their cognitive core issues more directly and to work on them in an experiential way.

We have described above the theoretical framework for our new embodied cognitive behavioral therapy approach to work on the dysfunctional schema, and how to incorporate embodied techniques in this approach’s three modules: body focus, emotional field, and interaction focus. For each of these three modules we portrayed examples of empirically proven embodiment techniques which can be used as bottom-up strategies to enable the efficient treatment of concrete problematic situations through the conscious use of the body. Module 1, the “body focus” is used for helping the patient to learn to accurately perceive, identify and give meaning to bodily signals and interoception. The second module, “the emotional field” is used to address problematic situations. This module starts with imaginative exposure, which is a standard CBT practice. As the emotional event unfolds, attention is paid to the first two stages of emotional awareness (Table 1), such as body signals, action and movement impulses. A second aspect of embodiment is being utilized at this module: the bidirectional relationship between body and psyche. Empirically validated posture, movement and breathing patterns—we call them embodiment techniques—help to intensify certain emotions such as fear, anger, disgust, sadness, etc. and in particular to clarify the sequence of primary and secondary emotions (Hauke and Dall’Occhio, 2013; Hauke and Lohr, 2020). If the patient can already offer a conceptualization, it can be checked to see whether it is valid and can help to clarify the extent to which secondary emotions prevent the primary emotion. The third module, the interaction focus, relates to the organization of the relationship with others and the achievement of the patient’s goals. The emotions developed in the emotional field and their messages are once again made directly accessible using embodiment techniques and provide the basis for the development of action plans.

Incorporating these embodied techniques offers several advantages over using traditional CBT: Prelinguistic or hard-to-grasp aspects can be identified more easily before being processed verbally. It is also easier to work with clients who have limited access to their emotions. With the help of specific movement characteristics as embodiment techniques, distinguishable emotions such as anger, sadness, disgust, shame, guilt, etc., can be triggered as well as intensified, and their problematic regulation can be examined *in vivo*. In addition, using specific zones in the space of the therapy-room allows the embodiment of problematic interactions, as well as of examining power and submissiveness, closeness and distance within interactions, etc. Directly experiencing these processes on one’s own body in the protected space of therapy enables patients to feel and process primary and secondary emotions which arise in problematic situations, and allows faster and deeper insights than would be possible with conversations alone. Finally, the vitalizing power of emotions is used to create coherent action plans and successful interactions.

Our approach has also some limitations: The application of our method requires that a well-established, supportive therapeutic relationship can be established. Acute psychiatric illnesses such as psychotic illnesses, bipolar disorders, etc. are therefore excluded. As our embodiment techniques require a bidirectional connection between mind and body, there may be limitations here if patients suffer from corresponding physical problems, such as chronic muscle tension with accompanying pain and restricted movement. We find chronic postural problems, for example, when patients can no longer fully straighten up, as we sometimes encounter in the case of long-term depressive illnesses. In many cases, suitable accompanying measures support the mode of operation of bidirectionality.

In sum: our embodied CBT method as described in details above, provides a well-structured experience that patients can reapply in difficult day-to-day situations to regulate emotions.

## Data availability statement

The original contributions presented in the study are included in the article/supplementary material, further inquiries can be directed to the corresponding author.

## Author contributions

GH: Writing – review & editing, Writing – original draft, Methodology, Conceptualization. CL-B: Writing – review & editing, Writing – original draft, Methodology, Conceptualization. TS: Writing – review & editing, Writing – original draft, Methodology, Conceptualization.
